# Association between anti‐herpetic medications and dementia risk: A systematic review and meta‐analysis

**DOI:** 10.1002/alz.085524

**Published:** 2025-01-09

**Authors:** Syuan‐Ting Chang, Yi‐ Fang Chuang

**Affiliations:** ^1^ School of Medicine, National Yang Ming Chiao Tung University, Taipei Taiwan; ^2^ National Yang Ming Chiao Tung University, Taipei, NA Taiwan

## Abstract

**Background:**

Previous research established a potential relationship between human herpesvirus (HHV) infections and increased risk of dementia. However, whether use of antiherpetic medications may lower dementia risk was not clear. Thus, this systematic review and meta‐analysis of cohort studies aimed to investigate the associations between anti‐herpetic medications and dementia risk.

**Method:**

This study was registered in PROSPERO (CRD42022368318) and adhered to PRISM guidelines. Databases from inception to October 2023 were extensively searched to identify cohort studies investigating the association between oral antiherpetic medications (ie. acyclovir, famciclovir, ganciclovir, valacyclovir, and valganciclovir) and dementia risk in non‐demented older adults 50 years old. Pooled Hazard Ratios (HR) were analyzed using a random‐effects model in STATA 18. Subgroup analysis was performed to explore potential sources of heterogeneity.

**Result:**

Twelve studies with 9,612,885 older adults assessing the impact of antiherpetic medications on dementia risk were included in our analysis. The meta‐analysis was divided into three comparison groups based on the study population setting: treated vs. untreated (regardless of infection diagnosis), diagnosed with and treated for infection vs. diagnosed but untreated, and diagnosed with and treated for infection vs. no diagnosed and no treatment. Our results showed that antiherpetic medications significantly reduced the risk of dementia by 12% (aHR = 0.88, 95% CI: 0.83‐0.93), 27% (aHR = 0.73, 95% CI: 0.65‐0.82), and 14% (aHR = 0.86, 95% CI: 0.77‐0.96) in each comparison group. Additionally, significant subgroup effect was found when considering severity of herpesvirus infection.

**Conclusion:**

This is the first systematic review and meta‐analysis of cohort studies to show that antiherpetic medications had significantly protective effect on dementia, particularly in those with severe herpesvirus infections. Our findings provide valuable insights for effectiveness of medical interventions and the possible mechanism of dementia. Future research should focus on clinical trials to comprehensively understand the causal effects of this association and the underlying mechanisms.

